# Optimized preoperative determination of nerve of origin in patients with vestibular schwannoma

**DOI:** 10.1038/s41598-021-87515-1

**Published:** 2021-04-21

**Authors:** Torsten Rahne, Stefan K. Plontke, Laura Fröhlich, Christian Strauss

**Affiliations:** 1 Department of Otorhinolaryngology, Head and Neck Surgery, University Hospital Halle (Saale), Martin Luther University Halle-Wittenberg, Halle (Saale), Germany; 2 Department of Neurosurgery, University Hospital Halle (Saale), Martin Luther University Halle-Wittenberg, Halle (Saale), Germany; 3 Universitäts-HNO-Klinik, Ernst-Grube-Str. 40, 06120 Halle (Saale), Germany

**Keywords:** Anatomy, Nervous system, Peripheral nervous system, Sensory systems, Medical research, Translational research

## Abstract

In vestibular schwannoma (VS) patients hearing outcome and surgery related risks can vary and depend on the nerve of origin. Preoperative origin differentiation between inferior or superior vestibular nerve may influence the decision on treatment, and the selection of optimal treatment and counselling modalities. A novel scoring system based on functional tests was designed to predict the nerve of origin for VS and was applied to a large number of consecutive patients. A prospective, double blind, cohort study including 93 patients with suspected unilateral VS was conducted at a tertiary referral centre. Preoperatively before tumor resection a functional test battery [video head-impulse test (vHIT) of all semicircular canals (SCC)], air-conducted cervical/ocular vestibular evoked myogenic potential tests (cVEMP, oVEMP), pure-tone audiometry, and speech discrimination was applied. Sensitivity and specificity of prediction of intraoperative finding by a preoperative score based on vHIT gain, cVEMP and oVEMP amplitudes and asymmetry ratios was calculated. For the prediction of inferior vestibular nerve origin, sensitivity was 73% and specificity was 80%. For the prediction of superior vestibular nerve origin, sensitivity was 60% and specificity was 90%. Based on the trade-off between sensitivity and specificity, optimized cut-off values of − 0.32 for cVEMP and − 0.11 for oVEMP asymmetry ratios and vHIT gain thresholds of 0.77 (anterior SCC), 0.84 (lateral SCC) and 0.80 (posterior SCC) were identified by receiver operator characteristic curves. The scoring system based on preoperative functional tests improves prediction of nerve of origin and can be applied in clinical routine.

## Introduction

Cochleovestibular schwannomas, usually termed as ‘vestibular schwannomas’ or ‘acoustic neuromas’, are benign tumors that most often develop in the human internal auditory canal and the cerebellopontine angle, and sometimes in the labyrinth^1^. Formerly thought to originate from Schwann cells in the glial-Schwannian transitional zone of the vestibulocochlear nerve, cochleovestibular schwannomas do in fact arise anywhere along the eighth cranial nerve^2–4^. The majority of cochleovestibular schwannomas present in the internal auditory canal (IAC) with or without extension to the cerebello-pontine angle (CPA). In these locations, they most often arise from the superior (SVN) or inferior vestibular nerve (IVN)^5–7^, while for the rarer intralabyrinthine schwannomas the cochlea is the site of origin in at least 50% of the cases^8^. This article focuses on the “classical” vestibular schwannoma (VS) in the IAC/CPA.

Patients with VS often experience early auditory symptoms, such as hearing loss and tinnitus and vestibular dysfunctions can occur with increasing tumor size^2,9^. Untreated VS will potentially destroy functional hearing within a couple of years^10^.

Surgical VS treatment not only aims on removing the tumor but also preserving facial nerve function and auditory function—the latter, if still present. Numerous factors influence hearing outcome. Besides tumor size and preoperative hearing status^6,11–15^, hearing preservation is reported to be more likely if the tumor arises from the SVN^11,16,17^. This is thought to be due to a closer anatomical relationship between the IVN, the cochlear nerve and the internal auditory artery. Dissecting a tumor arising from the IVN may involve a greater risk for compromising blood supply and traumatizing the cochlear nerve directly^16,17^. Knowledge about the nerve of origin could assist the surgeon in the intraoperative assessment of pathological anatomy, and facilitate better tumor removal. Preoperative differentiation of the nerve of origin may influence the decision on treatment, and the selection of optimal treatment and counselling modalities^18^.

Vestibular function is dependent on IVN and SVN integrity. However, it is not in the focus of a recent guideline on the diagnosis and treatment of VS^2^. It has been shown that vestibular function test results for the semicircular canals and the otoliths potentially allow to differentiate between IVN and SVN functional deficits and thus the respective VS origin^18,19^. Semicircular canal function can be measured with video head-impulse testing (vHIT)^20,21^. Based on the vestibular-ocular reflex, head and eye velocities are recorded simultaneously during and after a head impulse. Based on which of the canals are showing pathological results, an allocation to IVN and SVN deficits is possible^22–25^.

The cervical vestibular-evoked myogenic potential (cVEMP) test mostly evaluates saccular function while the ocular vestibular-evoked myogenic potential (oVEMP) test assesses mostly utricular function^26–29^. Since SVN and IVN integrity are prerequisites for assessment of otolith function, cVEMP and oVEMP recordings could conceivably differentiate between SVN- and IVN-mediated disturbance^25,29–33^.

Preoperative VEMP studies alone predict the nerve of origin inconsistently^34^, and a combination with caloric results did not show a correlation^35^. In a preliminary study, we reported a scoring algorithm integrating vHIT, cVEMP and oVEMP results to differentiate between tumor origin from IVN or SVN confirmed by intraoperative findings^18^. In that study, the score predicted tumor origin correctly in four of five consecutive VS patients.

This study aims on applying the preoperative scoring algorithm based on vHIT, cVEMP and oVEMP results in a large cohort of consecutive patients with VS surgery including those five of the initial study^18^ and to compare the predictions with the intraoperative surgically assessed tumor origin.

## Methods

### Study design and population

In a tertiary interdisciplinary referral centre at a university hospital, we conducted a double-blind clinical diagnostic study. We assessed eligibility of all consecutive adult patients undergoing primary surgery for VS treatment between January 2016 and March 2020. The study was approved by Ethical committee of the Medical Faculty of the Martin-Luther-University Halle-Wittenberg (approval number 2017-103) and conducted in accordance with the Declaration of Helsinki. Informed consent was obtained from all participants for being included in the study.

#### Preoperative testing

Patients underwent audiological and vestibular tests 1 day before surgery as part of the routine clinical protocol. Routine otomicroscopic evaluation of the external ear canals and the tympanic membranes were performed prior to collecting pure-tone audiogram including air-conduction hearing thresholds for frequencies of 0.125–8 kHz; and bone-conduction hearing thresholds for frequencies of 0.25–6 kHz. Word recognition score (WRS) was determined using the Freiburg speech test. Lists of monosyllabic words and multisyllabic numbers at various sound pressure levels (SPL) were presented with headphones. If a WRS of 100% was not achieved at 65 dB SPL, sound pressure was increased to a maximum of 100 dB SPL and the maximum percentage of monosyllables understood (WRS_max_) was determined. Hearing was classified according to^12,36^, adapted for German speech recognition tests^37^. Audiological assessments were conducted using an AT900 audiometer (Auritec, Hamburg, Germany).

To evaluate the vestibulo-ocular reflex (VOR), the head-impulse test was performed with a vHIT system (GN Otometrics, Taastrup, Denmark) according to Rahne et al.^18^. A light (60 g) and high-speed (250 Hz) eye camera with a built-in calibration laser was connected to the patient, enabling identification of overt and covert saccades. The head was quickly and unpredictably turned through 10°–20° angles in the horizontal, the LARP or RALP planes, to facilitate testing of the corresponding semicircular canals (LARP: left anterior-right posterior; RALP: right anterior-left posterior). We evaluated the absolute mean VOR gain between eye and head movement of the affected and non-affected sides. The appearance of covert and overt saccades after head impulses was analysed subjectively^18^. Artifacts due to methodological errors were avoided during the vHIT recording. Two experienced investigators analysed the recordings. If less than five clear covert or overt saccades could be discerned the respective SCC was labelled as free of saccades.

Air-conducted cVEMP were measured using the Eclipse Platform (Interacoustics, Copenhagen, Denmark), with surface electrodes placed on the upper half of the ipsilateral sternocleidomastoid muscles, a reference electrode on the mastoid, and the ground electrode on the forehead. An insert earphone was used to apply monaural acoustic stimulation as tone bursts of 500 Hz (rise/fall time, 2 ms; plateau time, 1 ms; 100 dB nHL)^18,38^. The stimulus rate was 5.1 Hz. For every presentation, at least 200 stimuli were averaged. After excluding a conductive hearing loss, the patients took a seated position and were instructed to rotate their heads toward the non-stimulated ear. Measuring the electromyogram (EMG) amplitude and delivering acoustic feedback to the patient maintained constant muscle tension. The first positive–negative peak (p13–n23) of the averaged EMG was defined as the cVEMP amplitude.

Air-conducted oVEMP were measured by placing the electrodes directly underneath the patients’ contralateral eyes, and applying acoustic stimuli to the ipsilateral ear. The ground electrode was placed on the forehead. The patients were asked to look maximally upwards while hearing the tones. The first negative–positive peak (n10–p15) of the averaged EMG was defined as the oVEMP amplitude^18^. Other stimulation and recording parameters were as described above for the cVEMP recordings.

We calculated the asymmetry ratios (AR) of the cVEMP and oVEMP amplitudes (AR_cVEMP_ and AR_oVEMP_, respectively) between the tumor-affected side and the non-affected side, using the ipsilateral (IA) and contralateral (CA) amplitudes at 100 dB nHL: AR = (IA − CA)/(IA + CA)^39^.

The nerve of origin was predicted by using the scoring system according to Rahne et al.^18^ (Table 1). A gain or asymmetry ratio smaller than the threshold levels was defined as pathologic. An allocation to either the anterior or lateral semicircular canals contributed one point to the SVN, while a pathological gain allocated to the posterior semicircular canal contributed two points to the IVN. Saccade occurrence allocated to either the anterior or lateral semicircular canals contributed one point each to the SVN, while saccade occurrence allocated to the posterior semicircular canal contributed two points to the IVN. Pathological cVEMP or oVEMP asymmetry ratios contributed four points to the IVN or SVN, respectively. Due to the increased weight for the posterior semicircular gain, the scoring was balanced between both nerve branches, with the potential to achieve a total score of eight.

**Table 1:** Scoring thresholds for determination of the most affected nerve of origin.

Assessment	Objective	Threshold	Scoring	
			IVN	SVN
**vHIT**				
Anterior semicircular canal	Gain	< 0.7	0	1
	Saccades	Present	0	1
Lateral semicircular canal	Gain	< 0.8	0	1
	Saccades	Present	0	1
Posterior semicircular canal	Gain	< 0.7	2	0
	Saccades	Present	2	0
**VEMP**				
Cervical	AR_cVEMP_	< − 0.36	4	0
Ocular	AR_oVEMP_	< − 0.36	0	4
Maximum score:			8	8

#### Surgery

Prior to surgery tumors were categorized according to the Koos classification^13,40^. With patients in the supine position and under total intravenous anaesthesia, tumor resection was performed via a retrosigmoid approach using facial and cochlear nerve monitoring. All operations were performed with the goal of preserving facial, cochlear, and remaining vestibular nerve function. During surgery, at least one experienced neurosurgeon (CS) who was blinded to the diagnostic results identified the nerve of VS origin. Criteria were adherence of the schwannoma to either vestibular nerves within the internal auditory canal and difficulties of dissection tumor from the respective vestibular portions, or in schwannomas with the fundus free of tumor, visual identification of the tumor origin. If the fundus is obliterated the position of the transverse crest at the fundus as localised with the rectangular nerve hook is helpful. In those tumors arising from the superior vestibular nerve the transverse crest is identified at a more caudal position due to the tumor growth pattern and vice versa. In isolated cases an endoscope was used. If the tumor’s origin could not be unambiguously determined, then the most likely nerve of origin was selected and reported to the data evaluation team. The diagnostic investigators were blinded to intraoperative findings.

### Study outcomes and statistical analysis

The primary outcomes were the sensitivity and specificity of prediction of intraoperative finding by a preoperative score. The Koos grade was recorded and used for stratification. According to Rahne et al.^18^, the scores for prediction of nerve origin were calculated based on amplitudes of the cVEMPs and oVEMPs, as well as the vHIT gain and occurrence of saccades. The nerve with the higher score was assumed to be most affected and was, thus, the presumptive nerve of origin.

The confusion matrix was calculated for IVN and SVN including sensitivity, specificity, positive and negative predictive values and Matthews correlation coefficient^41^. A multiple logistic regression was calculated to predict tumor origin based on vHIT gains, vHIT saccades, cVEMP AR and oVEMP AR. Receiver operating characteristic (ROC) curve analysis was performed for the primary outcome parameters including calculation of true positive, true negative, false negative and true negative rates for both, IVN and SVN nerve origin. Sensitivity and specificity were computed. Based on the results an optimized combination of predictive outcome was hypothesized using Youden’s index (true positive rate plus false negative rate^42^). The index *J* is an ROC parameter (*J* = sensitivity + specificity − 1) with values between 0 and 1. A large index graphically represents a large distance of the ROC curve from chance level (diagonal line) that is equivalent to a good discrimination performance. The maximum value of the index was used as criterion for selecting the optimum cut-off values of the diagnostic tests. All statistical analyses was performed using the SPSS software version 25 (IBM, Ehningen, Germany).

## Results

**Figure 1:**
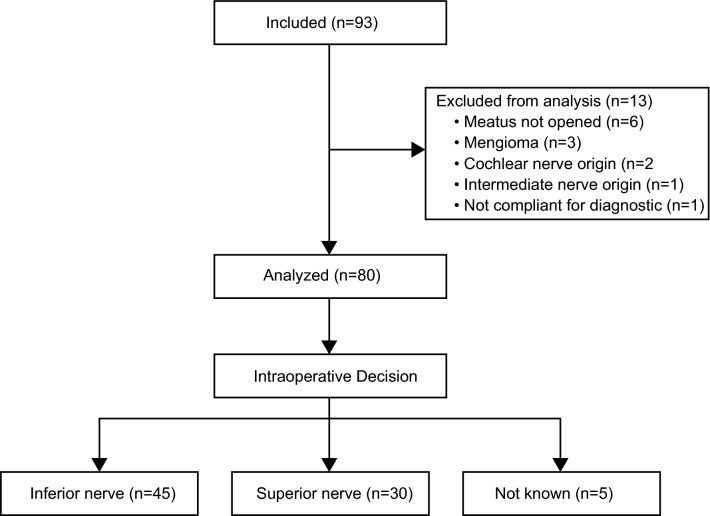
Flow chart of included excluded and analysed patients, as well as distribution of intraoperative decisions regarding nerve of origin.

A total of 93 patients undergoing surgery for VS treatment were included in the study (mean age 51.6 years, range 18–77 years; 28 male, 65 female). Demographic details are shown in Table 2. According to the Koos classification, tumors were diagnosed as stage I [13 patients (14%)], II [28 patients (30%)], III [31 patients (33%)], or IV [21 patients (23%)]. All included patients underwent surgery for tumor removal. After surgery, 13 patients were excluded from further analysis (see Fig. 1). Complete resection was achieved in all remaining cases.

**Table 2:** Demographic and audiological details of tumor patients.

Koos grade	n	Male/female (n)	Mean age (SD)/years	Right/left (n)	4PTA (SD)/dB HL		Mean WRS_65_ (SD)	Mean	Median hearing class (95% CI)	
					Ipsilateral	Contralateral		WRS_max_ (SD)	AAO-HNS^a^	GR
Total	80	26/54	51.5 (12.7)	42/38	49.4 (31.7)	17.1 (10.7)	49 (39)	70 (36)	2 (2.1; 2.6)	2 (2.1; 2.6)
I	12	3/9	48.1 (14.4)	8/4	35.3 (25.1)	15.5 (10.8)	59 (41)	81 (24)	1 (1.1; 2.8)	1 (1.1; 2.4)
II	26	6/20	53.9 (13.1)	14/12	45.8 (27.3)	18.8 (10.2)	53 (37)	79 (29)	2 (1.8; 2.6)	2 (1.8; 2.6)
III	25	10/15	52.1 (11.2)	12/13	52.5 (32.7)	16.6 (11.9)	45 (39)	69 (36)	2 (1.9; 2.9)	2 (1.8; 2.8)
IV	17	7/10	49.1 (13.3)	8/9	60.3 (38.1)	16.6 (10.3)	43 (42)	53 (46)	3 (2.1; 3.4)	3 (2.1; 3.9)

^a^1 = A, 2 = B, 3 = C, 4 = D; *SD* standard deviation, dB *HL* decibels hearing level, *GR* Gardner & Robertson, *4PTA* pure tone average at 0.5, 1, 2, 4 kHz, *WRS* word recognition score at 65 dB sound pressure level, *WRS*_*max*_ maximum WRS.

Figure 1 shows the intraoperative diagnosis of 45 (56%) patients with an IVN nerve origin and 30 (38%) patients with an SVN origin. In five (6%) patients, the surgeon could not make a clear decision. Applying the scoring system of Rahne et al.^18^, the preoperatively determined nerve of tumor origin was the SVN in 23 (29%) patients, the IVN in 40 (50%) patients, and 17 (21%) decisions were indifferent.

**Figure 2:**
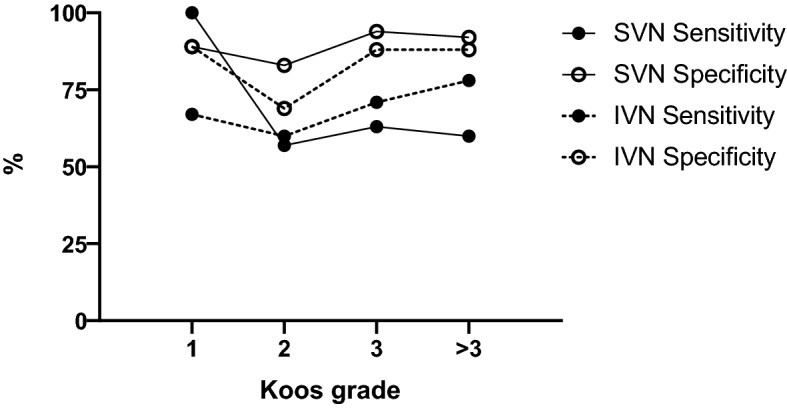
Sensitivity and specificity of the nerve of origin prediction by the scoring system stratified by Koos grade.

Table 3 shows the confusion matrix for both nerves of origin. For the prediction of IVN origin sensitivity was 73% and specificity was 80%. For the prediction of SVN origin sensitivity was 60% and specificity was 90%. Matthews’s correlation coefficients were 0.529 for IVN and 0.535 for SVN. Figure 2 shows the sensitivity and specificity stratified by the Koos grade. Sensitivity and specificity did not vary very much across the subgroups.

**Table 3:** Confusion matrix.

Superior vestibular nerve (SVN)					
		Surgical finding		Predictive value	
		SVN	not SVN	Positive	Negative
**Predicted origin**	SVN	18	5	0.78	
	Not SVN	12	45		0.71
	Sensitivity	0.60			
	Specificity		0.90		

Inferior vestibular nerve (IVN)					
		Surgical finding		Predictive value	
		IVN	not IVN	Positive	Negative
**Predicted origin**	IVN	33	7	0.83	
	Not IVN	12	28		0.46
	Sensitivity	0.73			
	Specificity		0.80

Table 4 shows the logistic regression analysis of SVN nerve of origin prediction. A significant regression was found for the compound model [χ^2^ (8) = 46.0, *p* < 0.001] with a Nagelkerke *R*^2^ of 0.625 which reflects a very strong effect. vHIT gains and occurrence of saccades for the anterior and lateral SCC as well as cVEMP ARs were significant predictors of the tumor origin.

**Table 4:** Logistic regression analysis of superior nerve of origin prediction.

Predictor	β	Standard error of β	Wald's χ^2^	*df*	*p*	*e* ^β^	95% confidence interval of *e* ^β^	
Constant term	− 1.814	2.331	0.605	1	0.437	NA	NA	NA
vHIT gain anterior SCC	6.817	3.455	3.893	1	**0.048**	913.283	1.047	797,013.613
vHIT gain lateral SCC	− 5.922	2.767	4.582	1	**0.032**	0.003	0.000	0.607
vHIT gain posterior SCC	3.021	1.811	2.781	1	0.095	20.508	0.589	714.075
vHIT Saccades anterior SCC	2.817	1.143	6.073	1	**0.014**	16.719	1.780	157.060
vHIT Saccades lateral SCC	− 2.855	0.886	10.376	1	**0.001**	0.058	0.633	24.198
vHIT Saccades posterior SCC	1.364	0.930	2.154	1	0.142	3.913	0.010	0.327
cVEMP AR	3.064	1.311	5.460	1	**0.019**	21.407	1.639	279.645
oVEMP AR	− 1.168	0.923	1.603	1	0.205	0.311	0.051	1.897

*AR* asymmetry ratio, *cVEMP* cervical vestibular evoked potentials, *NA* not applicable, *oVEMP* ocular vestibular evoked potentials, *SCC* semicircular canal.

**Figure 3:**
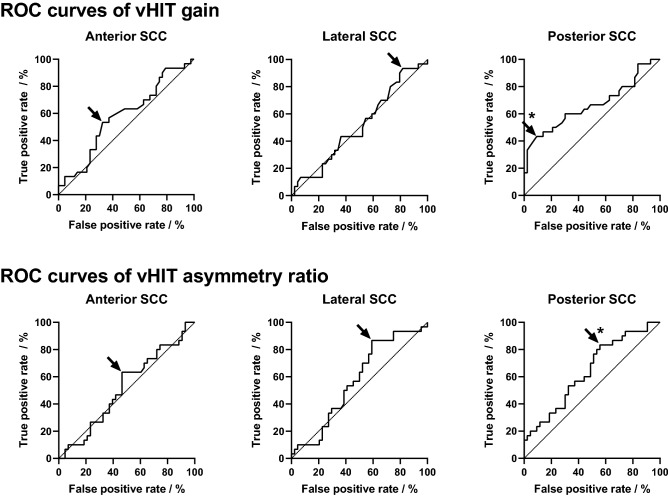
ROC curves for nerve of origin analysis based on vHIT gain and asymmetry ratio measures. The diagonal marks random classification. Asterisks (*) mark significant areas under curve (p < 0.05). Arrows mark the optimized cut-off value.

**Figure 4:**
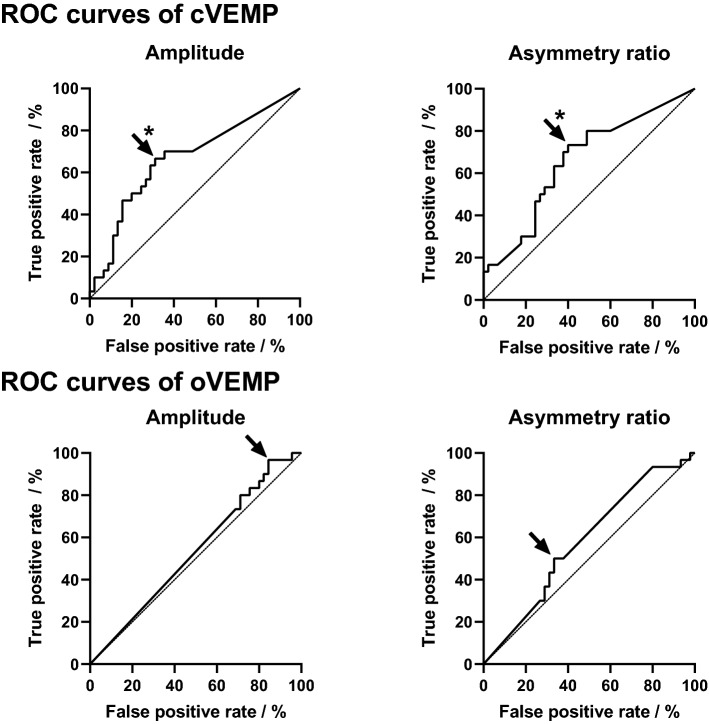
ROC curves for nerve of origin analysis based on cVEMP and oVEMP amplitude and asymmetry ratio measures. The diagonal marks random classification. Asterisks (*) mark significant areas under curve (p < 0.05). Arrows mark the optimized decision value.

Figure 3 shows the ROC curves for the vHIT gain and asymmetry ratios for all semicircular canals. Figure 4 shows the ROC curves for the cVEMP and oVEMP amplitudes and the respective asymmetry ratios. The figures show the test characteristics for the used vHIT and c/oVEMP thresholds. For the vHIT gain and asymmetry ratio of the posterior SCC, cVEMP amplitude and cVEMP asymmetry ratio, the area under the curves revealed significant classification (*p* < 0.05). Based on the trade-off between sensitivity and specificity, i.e., a maximized Youden’s index, optimized cut-off values were identified as − 0.32 for cVEMP and − 0.11 for oVEMP asymmetry ratios. Optimized vHIT gain thresholds would be 0.77 (anterior SCC), 0.84 (lateral SCC) and 0.80 (posterior SCC).

The scoring system was optimized by including vHIT gain and VEMP asymmetry ratio. Applying the improved scoring system based on the optimized cut-off values, the preoperatively predicted nerve of tumor origin was the SVN in 27 (34%) patients, and the IVN in 42 (52%) patients, while 11 (14%) decisions were indifferent.

Table 5 shows the confusion matrix for both nerves of origin after cut-off value optimization. For the prediction of IVN, origin sensitivity was 76% and specificity was 77%. For the prediction of SVN origin, sensitivity was 70% and specificity was 88%. Matthews’s correlation coefficient was 0.523 for IVN and 0.594 for SVN.

**Table 5:** Confusion matrix based on optimized decision criteria.

Superior vestibular nerve (SVN)					
		Surgical finding		*Predictive value*	
		SVN	not SVN	*Positive*	*Negative*
**Predicted origin**	SVN	21	6	0.78	
	Not SVN	9	44		0.68
	*Sensitivity*	0.70			
	*Specificity*		0.88		

Inferior vestibular nerve (IVN)					
		Surgical finding		Predictive value	
		IVN	not IVN	Positive	Negative
**Predicted origin**	IVN	34	8	0.81	
	Not IVN	11	27		0.44
	Sensitivity	0.76			
	Specificity		0.77		

## Discussion

In this study, VEMP and vHIT measurements for assessing vestibular receptor function were used to predict the nerve of origin for VS in a large number of patients. The results show prediction of the nerve of tumor origin diagnosed intraoperatively well above chance level. Sensitivity was larger for IVN tumors than for SVN tumors. Vice versa, specificity was larger for SVN tumors.

The prediction was based on the scoring system by Rahne et al.^18^ including vHIT amplitudes and saccades as well as AR of cVEMP and oVEMP amplitudes comparing the affected side to the non-affected side. Thus, several factors could have limited the predictability.

VS could potentially affect both nerves, as observed in the current dataset. Thus, to determine the nerve of origin, we hypothesized that the nerve with the highest score is the nerve of tumor origin. In the majority of cases, an experienced surgeon was able to determine the nerve of origin intraoperatively. Therefore, the risk of intraoperative misjudgement was considered very low in our study.

Despite VS induced hearing loss is widely independent of the tumor size^43^, the disturbance of vestibular nerve function increases with tumor size^9^. Large tumors are more likely to affect both, IVN and SVN and thus the results of vHIT and VEMP. Thus, tumor size seems to be a limiting factor for the prediction power. In the current study, the majority of tumors were categorized to Koos stages 2 and 3. As medium-sized tumors demonstrate the highest gain asymmetry^44^, we would expect a slightly larger prediction power, if only medium sized tumors were assessed. However, sensitivity and specificity did not vary very much across the subgroups (Fig. 2).

It is also possible for the cochlear or intermediate nerve to be the nerve of origin of a tumor in the cerebellopontine angle, such that the presently evaluated method could be misleading in a minority of cases. However, the main objective in deriving a preoperative prediction of the nerve of origin was to gather information about the risk for hearing loss, which would be mitigated, if the tumor origin was in the SVN^17^. That prediction would be irrelevant for cases of VS originating in the cochlear nerve. In the current study, such patients were excluded to focus on the prediction method on separating IVN and SVN tumors only.

Hearing preservation is dependent on the tumor size and on other anatomical factors as position of the VS in relation to the IAC or enlargement of the IAC. Also excreted exosomes from the tumors may be cochleotoxic independent or additionally to the nerve of tumor origin^45,46^. Nevertheless, determining the tumor origin prior to surgery may lead to improved predictions for hearing preservation and could thus become useful in counselling patients.

There is still some variation regarding the critical values (cut-off values) used to classify reduced vestibular function as pathologic. The criteria applied in our presently evaluated scoring system depend on the use of generally accepted cut-off values for vHIT^47,48^ and VEMP^49,50^. The present results allow for separately analysing the applied outcome parameters to optimize the cut-off values to increase sensitivity and specificity.

Our results reveal a rather small discrimination power between IVN and SVN tumors by using vHIT amplitudes alone (Fig. 3). The best discrimination power was observed if the posterior SCC was assessed by vHIT. Despite vHIT overall shows a large sensitivity in diagnosing VS^44^, prediction of tumor origin with vHIT alone would not be precise enough.

A good prediction power was observed, when cVEMP responses were compared between the affected and unaffected sides (Fig. 4). The discriminatory power based on the oVEMP responses, however, was very small. Here, the use of more effective stimulation methods, e.g., a powerful bone vibrator may reduce the large number of patients with no air-conduction driven oVEMP responses. Since VEMP responses are reduced specifically in the affected nerve, a combined analysis based on cVEMP and (BC) oVEMP responses might provide a high sensitivity for the prediction of nerve of origin. Sensitivity of oVEMP could potentially be improved by including bone-conduction stimulation.

The prediction power of the scoring system might also be limited by the age dependence of VEMP responses. VEMP test in the elderly is consider unreliable due to loss of hair cells of saccular and utricular macula in subjects aged > 60 years. Therefore, in those patients absent VEMP responses cannot clearly be differentiated caused by either aging or pathological effect. We hypothesize that the introduced scoring system would be more reliable if applied only to patients aged < 60 years.

Since both vHIT and VEMP results gradually contribute to the SVN and IVN assessment, the scoring system provides a compound rating method that should reduce false negatives via added redundancy. Cut-off values categorize vestibular sensor function dichotomously and specifically for each sensor^21,32,51^ and were optimized by the results. The optimized values found were rather close to the previously used values. The sensitivity was improved for SVN and IVN tumors while the specificity was still large (Table 4).

## Conclusions

The results reported here suggest that a prediction of the nerve of origin of VS is possible using the applied scoring system with a high agreement between preoperative and intraoperative findings. It is proposed that small VS tumors correlate with disturbance or reduced integrity of either the SVN or IVN, as reflected by pathological function of the semicircular canals or otoliths, respectively. Larger tumors are likely to also affect the other vestibular nerve branch and the intermediate or cochlear nerves as well. The assessments methods are commonly available in skull base centres with neurotological diagnostics. Its application could potentially improve prediction of tumour origin and its implication for hearing preservation.
